# RTF: an R package for modelling time course data

**DOI:** 10.1093/bioinformatics/btae597

**Published:** 2024-10-09

**Authors:** Eva Brombacher, Clemens Kreutz

**Affiliations:** Institute of Medical Biometry and Statistics, Faculty of Medicine and Medical Center-University of Freiburg, 79104 Freiburg, Germany; Centre for Integrative Biological Signalling Studies (CIBSS), University of Freiburg, 79104 Freiburg, Germany; Spemann Graduate School of Biology and Medicine (SGBM), University of Freiburg, 79104 Freiburg, Germany; Faculty of Biology, University of Freiburg, 79104 Freiburg, Germany; Institute of Medical Biometry and Statistics, Faculty of Medicine and Medical Center-University of Freiburg, 79104 Freiburg, Germany; Centre for Integrative Biological Signalling Studies (CIBSS), University of Freiburg, 79104 Freiburg, Germany

## Abstract

**Summary:**

The retarded transient function (RTF) approach serves as a complementary method to ordinary differential equations (ODEs) for modelling dynamics typically observed in cellular signalling processes. We introduce an R package that implements the RTF approach, originally implemented within the MATLAB-based *Data2Dynamics* modelling framework. This package facilitates the modelling of time and dose dependencies, and it includes the possibility of model reduction to minimize overfitting. It can be applied to experimental data or trajectories of ODE models to characterize their dynamics. Additionally, it can generate a low-dimensional representation based on the fitted RTF parameters of a set of time-resolved data, aiding in the identification of key targets of experimental perturbations.

**Availability and implementation:**

The R package RTF is available at https://github.com/kreutz-lab/RTF.

## 1 Introduction

Ordinary differential equation (ODE) models are currently the method of choice in systems biology for mathematically modelling the dynamics of signalling pathways within cells. However, ODEs have limitations, including the lack of analytical solutions and the need for large, complex models that require extensive experimental data to calibrate unknown parameters.

The retarded transient function (RTF) modelling approach has been introduced as a valuable complement to traditional ODE-based modelling (Kreutz 2020). Unlike mechanistic ODE models, the RTF is a phenomenological model that can describe individual observables without integrating all relevant components of a signalling process. It is specifically tailored to dynamic response curves that occur in ODE modelling and that are typically observed in cellular signalling pathways, which often feature new steady states after stimulation, monotonic behaviour or a single peak, and delayed responses. However, the RTF can also be applied to time course data unrelated to signalling, provided that the data exhibit these characteristics.

The RTF is designed for ease of use, offering fast and straightforward evaluation and accurate estimation of time- and dose-dependent system dynamics. Its parameters—amplitudes, rate constants, and time constants—are intuitively interpretable, enabling researchers without expertise in ODE modelling to analyse experimental biological data and make predictions about time and dose dependencies. The RTF models the time dependency of a signalling response through a sustained and a transient component, comprising three exponential functions and a nonlinear time transformation. It has also been extended to account for dose dependencies, i.e. the effects of varying treatment doses (Rachel *et al.* 2024). The clear interpretation of its parameters supports the characterization and understanding of signalling process dynamics.

We introduce an R package for RTF-based modelling, previously available only in MATLAB (Kreutz 2020) within the *Data2Dynamics* modelling framework (Raue *et al.* 2015), and more recently in Python (Höpfl *et al.* 2024). This package can be applied to experimental time course data as well as to data simulated from ODE models. To prevent overfitting, it includes model reduction through stepwise elimination of parameters.

ODE models often involve a large number of compounds and can quickly become complex, making it challenging to fully understand the dynamics of all included components, in particular if the dose dependency is considered in addition. To address this, our R package provides an additional feature: the ability to calculate a low-dimensional representation of signalling compound dynamics using uniform manifold approximation and projection (UMAP) applied to the fitted RTF parameters. For example, this low-dimensional representation can be generated based on RTF parameters fitted to trajectories for each pathway compound in an existing ODE model. The dynamics of the ODE model’s compounds is then reflected by the parameters of the RTF. In a UMAP plot based on these RTF parameters, each point represents the dynamics of a specific compound depicted in a low-dimensional representation. A shift of the position of those points can reveal qualitative differences in the dynamics of signalling compounds in response to experimental perturbations, such as gene knockouts.

Although the RTF is essentially an analytical function, offering it as part of an R package makes the equations and parameter bounds easily accessible and, provides a robust fitting procedure the RTF using nonlinear optimization (e.g. by calculating analytical derivatives), and provides additional functionalities, including model reduction and low-dimensional representation of signalling compound dynamics.

## 2 Retarded transient function

The RTF introduced by Kreutz (2020) describes time dependency as a combination of a sustained and a transient component and is defined as:


(1) $$\begin{array}{c} R(t,\theta^{R(t)})=\underset{\text{Sustained}\;\text{component}}{\underset{︸}{\mathrm{signSus}\times A\left(1-e^{-\alpha t}\right)}}+\\ \underset{\text{Transient}\;\text{component}}{\underset{︸}{\mathrm{signTrans}\times B\left(1-e^{-\beta t}\right)e^{-\gamma t}}}+b\;\;. \end{array}$$

**Figure 1.**
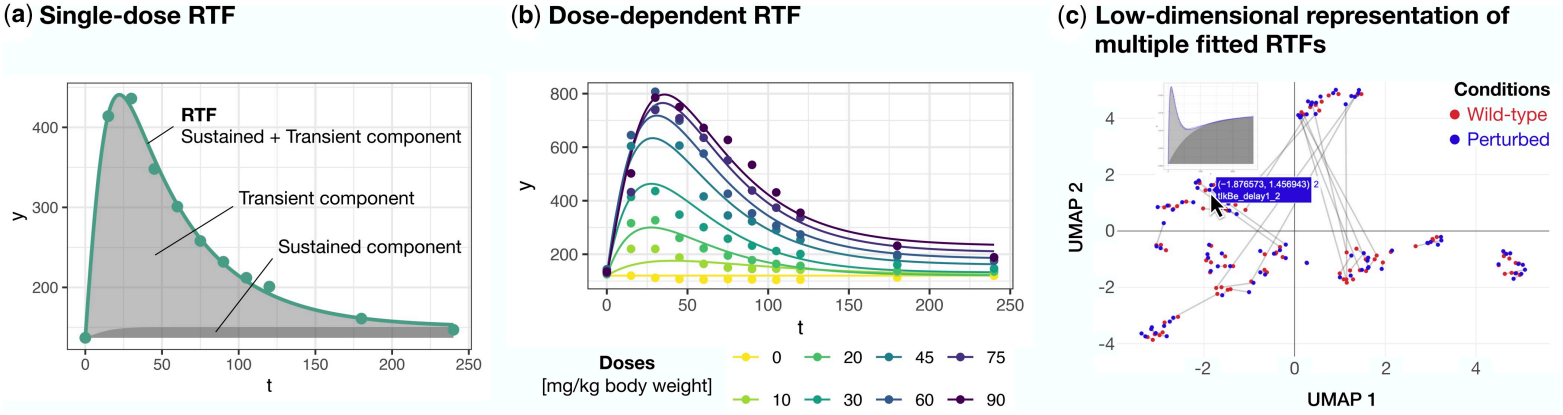
The three main applications of the RTF R package. (a) Time-resolved data modelled by the single-dose RTF. This subfigure is based on the dataset by Matsumoto et al. (2014) (*matsumoto*), where plasma amino acid profiles were measured after the ingestion of various branched-chain amino acids. The example shown depicts measured leucine plasma levels following the ingestion of 30 mg/kg body weight of leucine. (b) The dose-dependent RTF applied to time-resolved data generated at different doses. This subfigure, also based on the dataset by Matsumoto et al. (2014), illustrates measured leucine plasma levels across different ingested leucine concentrations. (c) Low-dimensional representation of multiple fitted RTFs. The results of multiple time course fits are visualized as a low-dimensional representation calculated from the RTF parameters, with each point representing one time course. This subfigure is based on the ODE model by Almaden et al. (2014), used here to generate time courses for the conditions ‘Wild-type’ and ‘Perturbed’ (*almaden*), shown in red and blue, respectively. To highlight qualitative changes in dynamics, time courses of the same molecular entity under the two conditions are connected by a line. If the time courses are too close to each other, the connecting line may not be visible. The cursor indicates the interactive selection of a time course, for which a plot of the dynamics, as well as the coordinates in the low-dimensional space and the name of the respective molecular entity, should be displayed.

Both components have their respective signs, *signSus* and *signTrans*, amplitudes *A*, *B*, and rate constants α, β, and γ (Fig. 1a). The explicit specification of *signSus* and *signTrans* enables external control over the signs, particularly when prior knowledge about the direction of the effects, i.e. activation or inhibition, is available.

These parameters are complemented by an offset parameter *b*. The argument *t* is a nonlinear transformation of the experimental time axis with a time-shift parameter τ, which accounts for the delayed response of pathway compounds:


(2) $$t(t_{\text{real}},\tau )={\;\text{log}\;}_{10}(10^{t_{\text{real}}\times 10/T}+10^{\tau })-{\;\text{log}\;}_{10}(1+10^{\tau })\;,$$

where $t_{\text{real}}$ represents the actual experimental measurement times, and *T* is the range of these measurement times.

To reduce overfitting, a custom model reduction procedure involving stepwise parameter elimination can be applied.

A modification of the terminology from the original publication (Kreutz 2020) was necessary to enhance readability, particularly because the original algorithm was extended to include dose dependencies (Rachel *et al.* 2024). Moreover, we had to replace the time scale parameters by the respective rates since we aimed for parameters to increase with increasing doses.

Hill equations are commonly used in pharmacological settings to describe how a response depends on the dose of a substance. To incorporate dose dependencies into the RTF approach and enable predictions for varying treatment doses, the dose-dependent parameters *A*, *B*, α, β, γ, and τ are described by Hill equations (Rachel *et al.* 2024):


(3) $$H(d)=M\frac{d^{h}}{K^{h}+d^{h}}\;.$$

This equation includes the dose *d*, along with three key parameters: the maximum value *M*, the half-maximal quantity *K* (corresponding to EC50), and the Hill coefficient *h*, which accounts for sigmoidality.

Specifically, to account for dose dependencies, *A* in Equation (1) is described by $M_{A}$, $h_{A}$, and $K_{A}$; *B* by $M_{B}$, $h_{B}$, and $K_{B}$; α by $M_{\alpha }$, $h_{\alpha }$, and $K_{\alpha }$; β by $M_{\beta }$, $h_{\beta }$, and $K_{\beta }$; γ by $M_{\gamma }$, $h_{\gamma }$, and $K_{\gamma }$; and τ by $M_{\tau }$, $h_{\tau }$, and $K_{\tau }$. We assume that *A*, *B*, α, β, and γ increase with increasing doses, while τ decreases.

The R package presented here incorporates the calculation of the RTF for time and dose dependencies, hereafter referred to as the ‘single-dose RTF’ and ‘dose-dependent RTF’.

## 3 Implementation

The primary purpose of the introduced package is to fit the RTF to experimental or simulated time course data.

The input data must be provided in the form of a table, called data frame in R, containing columns named ‘t’ (time) and ‘y’ (quantitative value), with ‘d’ (dose) and ‘sigmaExp’ (standard error of the experimental data) as optional columns. The ‘sigmaExp’ column can be included if the measurement error is known. In that case, it typically represents the standard error for mean values calculated from replicate measurements. If ‘sigmaExp’ is not provided, the error σ of the data points will be estimated along with all other RTF parameters.

A central structure for calculations within this package is the *optimObject*, a list that includes the input data frame (‘data’) along with several key components: a vector of default initial guesses for parameters (‘initialGuess.vec’) derived from the data, vectors for the lower (‘lb.vec’) and upper (‘ub.vec’) bounds, a vector of fixed parameters (‘fixed’), a Boolean vector (‘takeLog10’) indicating if log10 transformation is applied to the bounds, the mode (‘modus’), which can be ‘singleDose’ or ‘doseDependent’, and a vector of fitted parameter values (‘fitted’). By applying the *RTF()* function, the list is expanded to include the results from *stats::optim()* (R Core Team 2024) for all initial guesses (‘optimResults’), the *stats::optim()* result of the best fit (‘bestOptimResult’), and the likelihood value of the best fit (‘value’).

The parameters of the fitted RTF model are jointly estimated using the maximum likelihood approach. Specifically,


(4) $$-2\ln(\text{Likelihood})=-2\sum \left(\ln\left(\frac{1}{\sigma \sqrt{2\pi }}\right)+\left(-\frac{\Delta^{2}}{2\sigma^{2}}\right)\right)$$

is minimized, where Δ denotes the vector of the differences between the experimental data points and the data points estimated by the RTF for specific parameter values. For increased speed and accuracy during optimization, the analytical derivative of the RTF is calculated rather than using a finite difference approximation.

The package implements several key functions:

- *RTF()* estimates the best-fit RTF parameters for the provided input data. It can be run in ‘singleDose’ (Fig. 1a) or ‘doseDependent’ (Fig. 1b) mode, depending on whether signalling data at multiple doses are available. All parameters are jointly estimated based on maximum likelihood and multi-start optimization is conducted to increase the chance of finding a global minimum. By default, the fitted RTF function *fittedRTF()* is saved to an .R file, allowing users to conveniently make predictions for specified time points (and doses for the dose-dependent RTF). If a user wishes to improve upon a previous result, it can be provided to the function *RTF()* via the ‘resOld’ argument. The function then complements the previous result with additional optimization runs, and returns the best fit over both old and new initial guesses.
- *modelReduction()* applies a model reduction procedure (Kreutz 2020) to the *RTF()* result, iteratively eliminating parameters that are not necessary to explain the data, as determined by likelihood ratio tests. This results in a model with a minimal number of parameters and reduces the risk of overfitting. Like in the case of the *RTF()* function, this model is saved as a function in an .R file by default.
- *lowDimensionalRTF()* calculates a low-dimensional representation of multiple fitted RTFs. Specifically, it estimates the RTF parameters for multiple time courses and generates a two-dimensional UMAP based on these parameters. If the dynamics of certain molecular species differ between conditions, this should be reflected in changes to the RTF parameters, and consequently, in shifts within the UMAP. The formation of clusters in the UMAP indicates that the dynamics of the molecular species within the respective cluster are similar based on the RTF parameters. This function generates the following plots: (1) UMAP plots colour-coded by metadata and cluster affiliation according to k-means clustering; (2) a plot showing the 25th and 75th quantiles and the median of the fitted parameters for each cluster; and (3) plots illustrating the dynamics of the time courses, separated by cluster, where the time courses are displayed both unscaled and scaled (to enable qualitative comparisons of dynamics within each cluster).
- *plotInteractiveUMAP()* generates an interactive UMAP plot based on the low-dimensional representation of multiple fitted RTFs, where the dynamics for each time course (represented by a point) are displayed upon mouseover. The example in Fig. 1c shows the UMAP for the NFκB pathway dynamics from the dataset by Almaden *et al.* (2014) (introduced below), comparing two different conditions, with the time courses of the same molecular species connected by a line.
- *plotData()* plots the input data. For dose-dependent datasets, data points are colour-coded according to the respective dose.
- *plotRTF()* visualizes the results of the *RTF()* function. Specifically, the best RTF fit is displayed alongside the experimental data points, with the sustained and transient components of the RTF also depicted, as shown in Fig. 1a for the single-dose RTF and in Fig. 1b for the dose-dependent RTF. Additionally, the sorted multi-start optimization results for the different initial guesses are visualized in a waterfall plot, where a plateau at the best likelihood value indicates the global optimum. Finally, histograms showing the distribution of parameter values across all initial guesses are generated. For the dose-dependent RTF, dose values are also plotted against the estimated parameter values.

Example datasets can be simulated using the *getSimData()* function with ‘singleDose’ or ‘doseDependent’ mode. Additionally, the following datasets are available in the R package:

- *matsumoto*: Experimental dataset by Matsumoto *et al.* (2014), where the plasma profiles of 21 amino acids were measured after ingesting 10–90 mg/kg body weight of branched-chain amino acids (leucine, isoleucine, or valine). Data is available for 11 different time points.
- *strasen*: Simulated dataset based on the cell class model by Strasen *et al.* (2018b), reflecting six signalling classes observed upon stimulation with 100 pM transforming growth factor (TGF) β1. For six cell classes, the time courses of 23 signalling proteins were simulated using JWS Online (Olivier and Snoep 2004, Strasen *et al.* 2018a). Data is available for 101 different time points.
- *almaden*: Simulated protein dataset based on the model of NF-κB-signalling in B cells introduced by Almaden *et al.* (2014). This time course dataset was generated using the implementation of this model in ‘Data2Dynamics’ (Raue *et al.* 2015) and contains simulated time course data for 91 molecular entities. The dataset includes two conditions: a genetic perturbation that prevents the processing of p100 to p52 (condition 2) and wild-type controls (condition 1), resulting in a total of 182 time courses. Data is available for 101 different time points.

Two basic examples of how to apply the RTF R package are given below:

# devtools :: install_github(“kreutz-lab/RTF”)

library(RTF)

# Example 1: RTF applied to a simulated single-dose dataset

data.singleDoseSim <- getSimData(modus = “singleDose”)

plotData(data = data.singleDoseSim)

optimObject.singleDoseSim <- RTF (

  df = data.singleDoseSim, modus = “singleDose”)

plot.singleDoseSim <- plotRTF (

  optimObject = optimObject.singleDoseSim,

  plotAllFits = FALSE)

prediction <- fittedRTF ( times = c(0, 1, 3, 4, 6, 12, 22))

# Example 2: RTF applied to the experimental dose-dependent dataset by  Matsumoto *et al.* (2014)(see  Fig. 1b)

matsumotoLeuLeu <- matsumoto [

  matsumoto$measuredAA == “Leu” &

    matsumoto$ingestedBCAA == “Leu”,]

data.doseDependentExp <-

  matsumotoLeuLeu[, c(“time”, “value”, “dose”)]

colnames(data.doseDependentExp) <- c(“t”, “y”, “d”)

plotData(data.doseDependentExp)

optimObject.doseDependentExp <- RTF (

  df = data.doseDependentExp, modus = “doseDependent”)

plot.doseDependentExp <- plotRTF (

  optimObject = optimObject.doseDependentExp,

  plotAllFits = FALSE)

prediction <- fittedRTF (

  times = c(0, 20, 100, 150, 300),

  doses = c(5, 25, 110))

## 4 Discussion

The R package introduced here makes the RTF approach for modelling time- and dose-dependent responses, typically observed in signalling pathway entities, accessible to the large R community in bioinformatics.

By applying the RTF, users can derive a small set of parameters that effectively characterize signalling dynamics, offering new opportunities for interpreting the effects of perturbations on these pathways. The package also enables the generation of two-dimensional representations that highlight compounds with similar dynamics, e.g. through clustering. Moreover, comparing multiple experimental or biological conditions within these representations can uncover shifts in dynamic behaviour, as evidenced by compounds transitioning from one cluster to another under perturbed conditions.

Looking ahead, the R package could be further enhanced to include the calculation of parameter uncertainties using the profile likelihood approach (Steiert *et al.* 2012, Kreutz *et al.* 2013).

## Data Availability

There are no new data associated with this article.
